# MSI2 regulates NLK-mediated EMT and PI3K/AKT/mTOR pathway to promote pancreatic cancer progression

**DOI:** 10.1186/s12935-024-03444-9

**Published:** 2024-08-03

**Authors:** Longping Huang, Jian Sun, Yuteng Ma, He Chen, Chen Tian, Ming Dong

**Affiliations:** 1 Department of Gastrointestinal Surgery, The First Hospital, China Medical University, Shenyang, 110001 China; 2 Department of Gastroenterology and Hepatology, The Fourth People’s Hospital of Shenyang, Shenyang, 110031 China

**Keywords:** Musashi2, NLK, PI3K/AKT/mTOR pathway, EMT, Pancreatic cancer

## Abstract

**Background:**

The incidence of pancreatic cancer is increasing by years, and the 5-year survival rate is very low. Our team have revealed that Musashi2 (MSI2) could promote aggressive behaviors in pancreatic cancer by downregulating Numb and p53. MSI2 also facilitates EMT in pancreatic cancer induced by EGF through the ZEB1-ERK/MAPK signaling pathway. This study aims to further explore the molecular mechanisms of MSI2-regulated downstream pathways in pancreatic cancer.

**Methods:**

In vitro and in vivo experiments were conducted to investigate the role and mechanism of MSI2 in promoting malignant behaviors of pancreatic cancer through regulation of NLK.

**Results:**

Genes closely related to MSI2 were screened from the GEPIA and TCGA databases. We found that NLK showed the most significant changes in mRNA levels with consistent changes following MSI2 interference and overexpression. The high correlation between MSI2 and NLK was also observed at the protein level. Multivariate analysis revealed that both MSI2 and NLK were independent adverse indicators of survival in pancreatic cancer patients, as well as join together. In vitro, silencing or overexpressing NLK altered cell invasion and migration, by regulating EMT and the PI3K-AKT-mTOR pathway. Silencing MSI2 reduced protein expression in the EMT and PI3K-AKT-mTOR pathways, leading to decreased cell invasion and migration abilities, while these effects could be reversed by overexpression of NLK. In vivo, MSI2 silencing inhibited liver metastasis, which could be reversed by overexpressing NLK. Mechanistically, MSI2 directly binds to the translation regulatory region of NLK mRNA at positions 79–87 nt, enhancing its transcriptional activity and exerting post-transcriptional regulatory roles. The analysis of molecular docking showed the close relationship between MSI2 and NLK in pancreatic cancer patients.

**Conclusions:**

Our findings elucidate the regulatory mechanisms of the MSI2-NLK axis in modulating aggressive behaviors of pancreatic cancer cells, which providing new evidence for therapeutic strategies in pancreatic cancer.

**Supplementary Information:**

The online version contains supplementary material available at 10.1186/s12935-024-03444-9.

## Background

The incidence of pancreatic cancer is increasing at a rate of 0.5–1.0% per year, and it is projected to become the second leading cause of cancer-related deaths in the United States by 2030 [1]. Due to its silent onset, pancreatic cancer is often diagnosed at a late stage when the surgery is no longer possible. Even among patients without distant metastasis who undergo curative surgical treatment, the five-year survival rate remains low [2]. Pancreatic cancer appear epithelial-mesenchymal transition (EMT), losing its epithelial characteristics and acquiring invasive stromal cell properties, leading to a highly malignant phenotype which is closely associated with poor prognosis in pancreatic cancer patients [3].

Musashi2 (MSI2) is an RNA-binding protein (RBP), originally discovered in fruit flies as a translational repressor, which regulates gene expression at the post-transcriptional level. MSI2 plays important roles in cell asymmetric division, stem cell maintenance, neural differentiation, hematopoietic stem cell expression, and regulation of the gastrointestinal system [3–7]. Previous studies have found that MSI2 is not only involved in the regulation of leukemia in the hematopoietic system but also aberrantly expressed and involved in the progression of solid tumors such as breast cancer, liver cancer, and oral squamous cell carcinoma [8–12]. Our previous research has shown that overexpression of MSI2 can promote the invasion and metastasis of pancreatic cancer cells by downregulating Numb [13]. In addition, MSI2 can promote chemoresistance and malignant biological behavior in pancreatic cancer by upregulating p53 [14]. MSI2 also promotes EMT induced by EGF in pancreatic cancer through the ZEB1-ERK/MAPK signaling pathway [3].

In this study, we screened the GEPIA databases to identify the most correlated 15 genes with MSI2 expression, and found a significant correlation between the expression of MSI2 and Nemo-like kinase (NLK). So we focused on investigating the regulatory role of MSI2 on NLK. NLK is a conserved serine/threonine protein kinase that regulates extracellular signal-regulated kinase/microtubule-associated protein kinase (Erks/MAPKs) and cyclin-dependent kinase (CDK) family members [15]. In 1994, NLK was reported that mutations in the nemo gene reduced the survival rate of fruit flies and caused abnormal head and eye development. In 1998, the mammalian homolog of nemo gene was cloned and named NLK [16]. NLK has been found to regulate multiple signaling pathways and participate in various biological processes [17–19]. Abnormal expression of NLK is closely associated with the occurrence and progression of tumors, with high expression in laryngeal cancer, lung cancer, and cervical squamous cell carcinoma [20–22], but with low expression in non-small cell lung cancer, breast cancer, and ovarian cancer [23–25]. Currently, there are no reports on the expression of NLK in pancreatic cancer and its relationship with prognosis. This study is the first to report the role of NLK in the progression of pancreatic cancer and confirms that MSI2 promotes pancreatic cancer development and affects prognosis by directly binding to NLK.

## Materials and methods

### Bioinformatics analysis

The 15 genes most closely related to MSI2 were screened using the GEPIA database (http://gepia.cancer-pku.cn/) and further confirmed in the TCGA database. In the TCGA-PAAD dataset, the correlation between MSI2 and NLK target genes was analyzed in 177 pancreatic cancer samples. The expression levels of MSI2 and NLK were used to calculate the correlation coefficient (r-value) and significance (p-value) based on Spearman correlation analysis. Functional enrichment analysis was conducted using KEGG analysis.

### Tissue samples and cell lines

90 paraffin-embedded pancreatic ductal adenocarcinoma samples were collected from surgical patients at the Department of Gastrointestinal Surgery of the First Hospital of China Medical University and the Department of gastroenterology and hepatology surgery of Shenyang Fourth People’s Hospital between 2011 and 2021. All data were accompanied by complete follow-up information. Human pancreatic cancer cell lines Capan-2, Panc-1, and Miapaca-2 were purchased from the Cell Bank of the Chinese Academy of Sciences (Shanghai, China).

### Construction of silenced/overexpressed cell lines

Lenti-cas9 and lenti-sgRNA were synthesized by Genechem. MSI2-sgRNA and sgRNA control were synthesized by GenePharma. NLK-shRNA and shRNA control were also synthesized by GenePharma. Lentiviral vectors for NLK overexpression (Lv-NLK), lentiviral vectors for MSI2 overexpression(Lv-MSI2) and corresponding control empty vectors (Lv-Vector) were synthesized by Genechem. Stable cell lines were constructed according to the instructions of the reagent manufacturers.

### Immunohistochemistry

Paraffin-embedded samples were cut into 4 μm thick sections. The sections were covered with 0.3% H_2_O_2_, subjected to high-pressure antigen retrieval for 3 min, blocked with 3% H_2_O_2_ and 10% goat serum for 30 min, and incubated with primary antibodies (Table S2) at 4℃overnight. The secondary antibodies were incubated together for 30 min, and the experiment was performed using 3,3’-diaminobenzidine (DAB) according to the manufacturer’s protocol. Negative controls were performed using isotype-matched antibodies. The immunoreactive score (IRS) was calculated as the staining intensity (SI) multiplied by the percentage of positive cells (PP). The SI score ranged from 0 to 3 (negative, weak, moderate, and strong), and the PP score ranged from 0 (<5%) to 4 (>75%) based on the positive staining area of the entire cancer. The final score ranged from 0 to 12, with a score greater than 6 indicating high expression.

### Western blot

Whole protein lysates were prepared from treated PC cell lines. The samples were electrophoresed in 10% SDS-PAGE and transferred to PVDF membranes. Standard immunoblotting was performed using specific antibodies and the ECL detection kit (Thermol Biotech, USA). The immunoimprinting were detected using a Bio-Rad imaging system, and ImageLab software was used for analysis.

### Quantitative real-time PCR

RNA was extracted using the TRIZOL reagent (Takara Bio). cDNA was generated using the Reverse Transcription Kit (Thermo Biotech Inc, USA). Real-time quantitative PCR was performed using the Light Cycler kit (Takara) on the Light Cycler 2.0. Primer sequences can be found in Table S1 (Shanghai Sangon Biotech). The quality of PCR products was determined by analyzing the melting curves after PCR. The expression levels were calculated using the 2^-ΔΔCt^ (relative quantification method).

### Invasion and migration assays

Cell migration assays were performed by seeding 2 × 10^^5^ cells in the upper chamber of a 24-well plate with serum-free medium. The lower chamber was filled with medium containing 10% FBS as a chemoattractant. After 24 h, migrated cells were fixed with 4% methanol and stained with 1% crystal violet (Sigma). Invasion assays were performed by coating the top of the membrane with Matrigel (BD Biosciences, USA) and following the same conditions. The final number of migrated and invaded cells was calculated by counting five randomly selected fields in each chamber at 40x magnification using a Nikon Microhot FX microscope.

### Molecular docking

The HDOCK online platform (http://hdock.phys.hust.edu.cn/) was used for molecular docking analysis of protein-protein or protein-nucleic acid interactions. Protein sequences were modeled using the SWISSMODEL server, and nucleic acid sequences were further modeled using the 3DRNA server. The protein and nucleic acid were then docked. The PyMOL (version 4.3.0) software was used to visualize the amino acid residues and bases involved in the interaction between the two docked molecules.

### Prediction of binding sites

The 197 bp sequence of the NLK gene translational regulatory region was analyzed, and input into the catRapid website to analyze its specific binding sites with the MSI2 protein (http://service.tartaglialab.com/update_submission/).

### RNA-immunoprecipitation

Pancreatic cancer cells transfected with NLK expression plasmids were sonicated, and the supernatant containing the protein-RNA complexes was collected. Immunoprecipitation was performed using MSI2 antibody and IgG antibody. DynaGreenTM Protein A/G Magnetic Beads were added to each group, and the antibody-antigen complex solution was mixed. The beads were separated, and purified RNA was extracted and reverse transcribed. Primers for RNA-immunoprecipitation were designed using “Primer Premier” software (see Table S5), and PCR and agarose gel electrophoresis were performed to analyze the differences between groups.

### RNA pulldown

Biotin-labeled wild-type and mutant RNA probes containing the NLK translational regulatory region(79-87nt) were synthesized(Shanghai Sangon Biotech). Three portions of 50 µl DynabeadsTM Streptavidin beads were added to 1.5 ml EP tubes. Group 1 and Group 2 were incubated with 50pmol of wild-type and mutant RNA probes, respectively, while Group 3 was used as a negative control without probes. The tubes were incubated at room temperature on a rocking shaker for 1 h (5r/min), and the magnetic beads were separated and the supernatant discarded. The pancreatic cancer cells were sonicated after three freeze-thaw cycles, and the supernatant was collected by centrifugation. The supernatant was added to the tubes and incubated overnight at 4℃ on a rocking shaker (5r/min). The beads were washed with 1× sample loading buffer, boiled for 10 min, and used for Western blot analysis of MSI2.

### Xenograft mouse model

Experimental animals were maintained by the animal care facility of China Medical University following the principles of the National Guidance for Animal Experimentation. A total of twelve female BALB/c nude mice (6–8 weeks old) were acclimated for one week. They were randomly divided into four groups: MSI2-Ctrl + Lv-Ctrl, MSI2-Sg + Lv-Ctrl, MSI2-Sg + Lv-NLK, and MSI2-Ctrl + Lv-NLK (*n* = 3 per group). Under sodium pentobarbital anesthesia, a horizontal small incision (1 cm) was made on the left flank, and the spleen was identified and exposed. Pancreatic cancer cells (2 × 10^^6^) mixed with pre-chilled PBS (100 µl) were slowly injected into the spleen’s lower pole. Three weeks later, all mice were euthanized. The number of liver metastases was counted, and specimens were collected for HE staining.

### Statistical analysis

Statistical analysis was performed using SPSS software version 20.0 (SPSS, Chicago, IL, USA). Differences in qRT-PCR, Western blot, Transwell assay, and liver metastasis counts were presented as means ± SE and compared using Student’s t-test. The expression of different proteins in IHC was compared using non-parametric tests. The correlation between MSI2 and NLK with clinicopathological parameters was analyzed using the chi-square test. COX’s regression analysis was performed to identify multiple risk factors. The relationship between target proteins in pancreatic cancer specimens was analyzed using Spearman correlation. Kaplan-Meier curves were used to estimate survival rates, and differences were analyzed using the log-rank test (*p*<0.05 was considered statistically significant).

## Results

### The expression of NLK is closely associated with MSI2

**Fig. 1:**
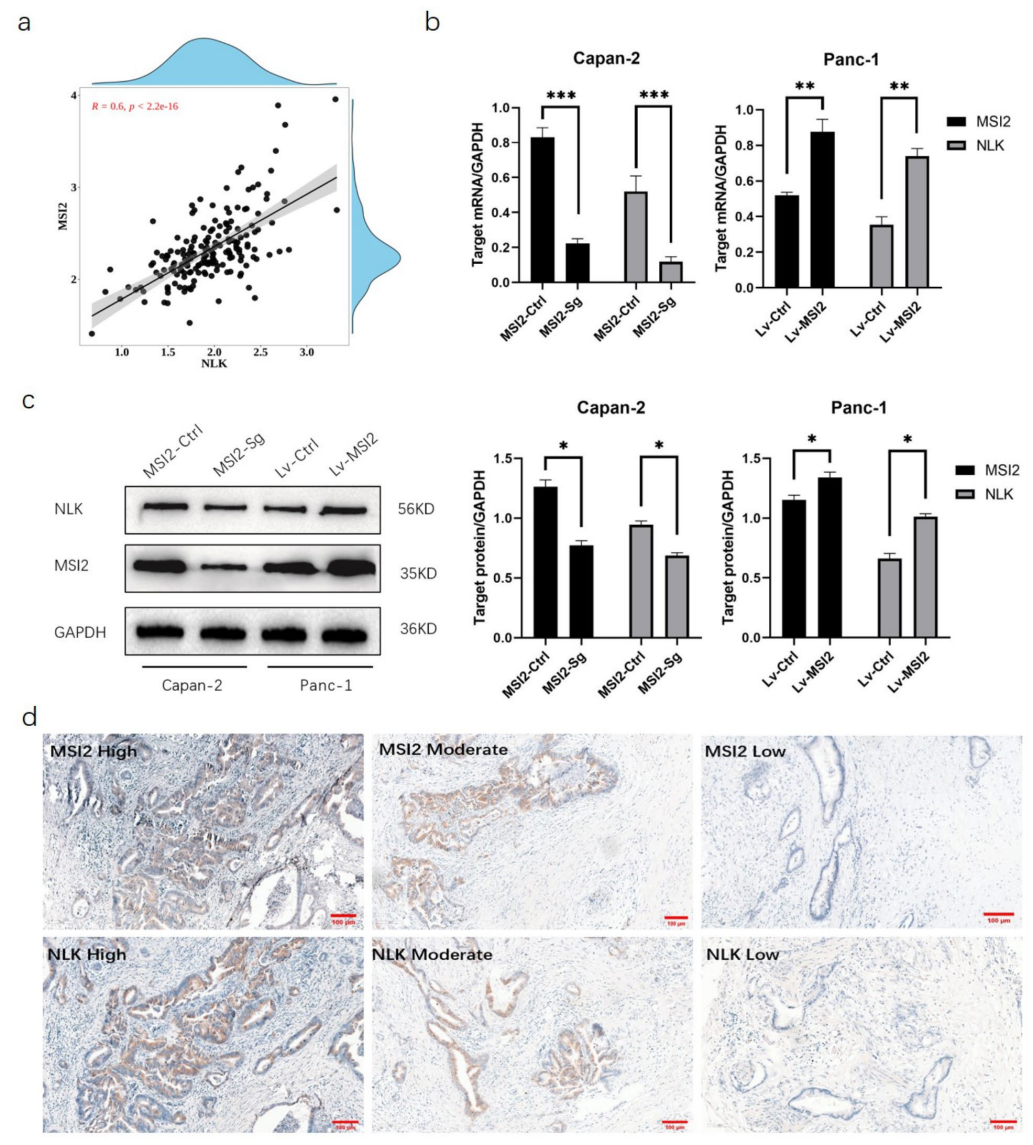
The expression of NLK is closely associated with MSI2. (**a**) Correlation analysis of MSI2 and NLK in TCGA database (**b**) qRT-PCR showed that the RNA level of NLK varied with the expression of MSI2. (**c**) Western Blot showed that the protein level of NLK varied with the expression of MSI2. (**d**) Immunohistochemistry showed that the expression of MSI2 and NLK was consistent in pancreatic cancer

Using the GEPIA database, we identified the top 15 genes that are closely correlated with MSI2 expression (Table S1). Screening of mRNA level changes in MSI2 knockdown stable cell lines established in Capan-2 cells and MSI2 overexpression stable cell lines constructed in Panc-1 cells by qRT-PCR, we selected NLK as the downstream gene of MSI2 for further experiments (Figure S1). In the TCGA-PAAD dataset, the expression levels of MSI2 and NLK were closely correlated (*r* = 0.60, *p*<0.05) (Fig. 1a). The mRNA levels of NLK showed the most significant changes in response to MSI2 knockdown and overexpression in pancreatic cancer cell lines, as observed in Capan-2 cells (MSI2-Ctrl VS MSI-Sg) and Panc-1 cells (Lv-Vector VS Lv-MSI2) (Fig. 1b). Western blotting also revealed consistent results at the protein level, with NLK expression decreasing with MSI2-Sg expression in Capan-2 cells and increasing with Lv-MSI2 expression in Panc-1 cells (Fig. 1c). These results indicate that MSI2 can regulate the expression of NLK. Immunohistochemical results showed that MSI2 is primarily expressed in the cytoplasm, while NLK is expressed in both the cytoplasm and the nucleus of pancreatic cancer cells, and there is close correlation between MSI2 and NLK expression (Fig. 1d). Spearman correlation analysis showed a positive correlation between MSI2 and NLK expression in the 90 pancreatic cancer tissues (*p*<0.001) (Table 1).

**Table 1:** Correlation analysis between MSI2 and NLK

		MSI2		*r*	*p*
		Low	High		
NLK	Negative	26	15	0.46	< 0.001
	Positive	9	40		

### Clinical significance of MSI2 and NLK expression in pancreatic cancer

**Fig. 2:**
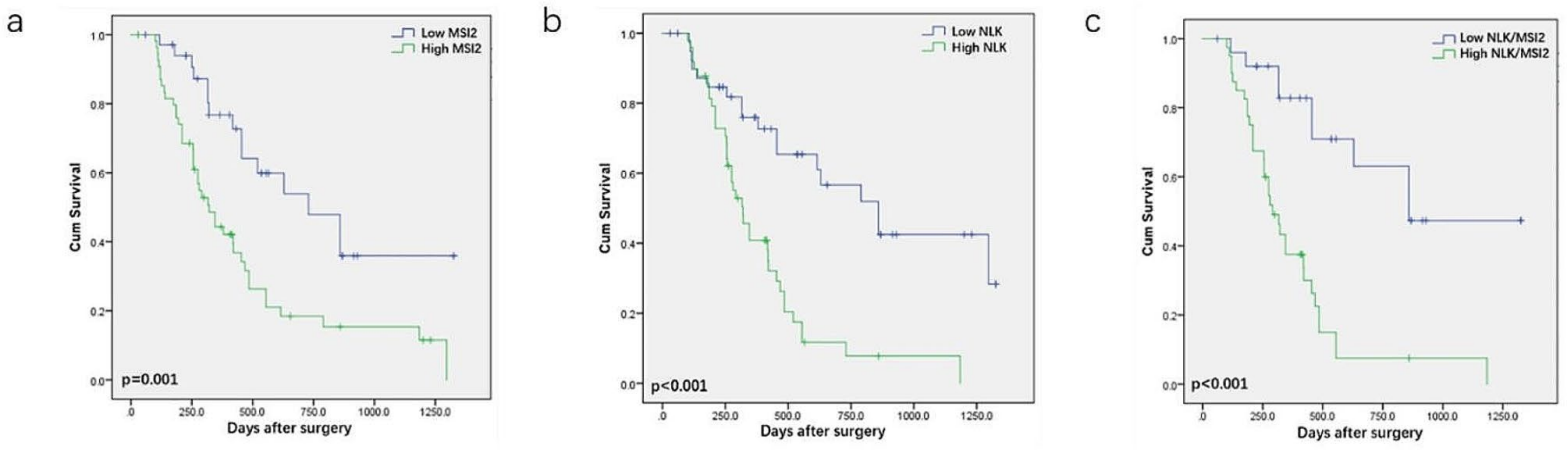
Relationship between the expression levels of MSI2 and NLK and survival in 90 PC patients. **a** Kaplan-Meier curve showed the relationship between the prognosis of PC patients and MSI2 expression level. **b** Kaplan-Meier curve showed the relationship between the prognosis of PC patients and NLK expression level. **c** Kaplan-Meier curve showed the relationship between the prognosis of PC patients and MSI2 and NLK expression levels

A correlation analysis of MSI2 and NLK with tumor clinicopathological features was conducted in 90 pancreatic cancer tissues. The results showed that high expression of MSI2 was positively correlated with tumor size (*p*=0.002), lymph node metastasis (*p*<0.001), UICC stage (*p*=0.006), and perineural invasion (*p*=0.03). Similarly, high expression of NLK was positively correlated with tumor size (*p*<0.001), lymph node metastasis (*p*<0.001), UICC stage (*p*=0.009), CA19-9 value (*p*=0.03), and vascular invasion (*p*=0.04) (Table 2). Survival analysis of the 90 patients revealed that patients with high MSI2 expression had significantly lower overall survival compared to patients with low MSI2 expression (*p*=0.001) (Fig. 2a), and patients with high NLK expression also had poor overall survival (*p*<0.001) (Fig. 2b). Furthermore, patients with both high MSI2 and NLK expression had significantly lower overall survival compared to patients with both low MSI2 and NLK expression (*p*=0.001) (Fig. 2c). Univariate and multivariate analyses of survival in postoperative pancreatic cancer patients revealed that the positive expression of MSI2 and NLK, as well as postoperative liver metastasis, were independent prognostic indicators for pancreatic cancer patients (Table 3).

**Table 2:** Correlation analysis of MSI2 and NLK expression with clinical data

Parameters	No.of patients	MSI2		*P*	NLK		*P*
		low	high		low	high	
Cases	90	35	55		41	49	
Ages(years)							
≤ 65		25	39	0.96	30	34	0.69
>65		10	16		11	15	
Gender							
Male		28	37	0.19	28	37	0.45
Female		7	18		13	12	
Tumor location							
Head		26	39	0.73	31	34	0.51
Body-tail		9	16		10	15	
Tumor size(cm)							
<2.5		18	11	0.002	21	8	< 0.001
≥ 2.5		17	44		20	41	
Differentiation							
Well		11	22	0.41	14	19	0.65
Moderate		24	33		27	30	
T stage							
T1 + T2		15	13	0.06	14	14	0.57
T3 + T4		20	42		27	35	
Lymph nodes metastasis							
N0(negative)		11	38	<0.001	2	47	<0.001
N1(positive)		24	17		39	2	
UICC stage							
I + IIA		30	32	0.006	34	28	0.009
IIB + III		5	23		7	21	
Pre-therapeutic CA19-9 level							
<37 U/ml		13	14	0.24	17	10	0.03
≥ 37 U/ml		22	41		24	39	
Perineural invasion							
Absent		32	40	0.03	36	36	0.09
Present		3	15		5	13	
Vascular permeation							
Absent		23	26	0.09	27	22	0.04
Present		12	29		14	27	
Postoperative Liver metastasis							
Negative		27	34	0.13	31	30	0.15
Positive		8	21		10	19	

**Table 3:** Univariate and multivariate clinicopathological analysis of survival for 90 pancreatic cancer patients after surgery

Parameters	Univariate analysis hazard ratio(95%CI)	*P*	Multivariate analysis hazard ratio(95%CI)	*P*
Age (<65/≥65 years)	1.40(0.77,2.52)	0.27		
Gender (male/female)	1.34(0.75,2.41)	0.32		
Tumor location (Head/Body-tail)	1.54(0.89,2.66)	0.12		
Tumor size (<2.5/≥2.5 cm)	1.71(0.94,3.12)	0.08		
well/poor and moderate Differentiation	1.50(0.86,2.61)	0.15		
T stage (T1 + T2/T3 + T4)	2.06(1.11,3.85)	0.02	1.43(0.73,2.82)	0.3
Lymph nodes metastasis(N0/N1)	0.42(0.24,0.74)	0.003	3.76(0.79,17.87)	0.1
UICC stage(I + IIA/IIB + III)	1.93(1.14,3.27)	0.02	1.28(0.71,2.27)	0.4
CA19-9 level (<37 U/ml/≥37 U/ml)	1.55(0.85,2.83)	0.15		
Perineural invasion (absent/present)	1.55(0.81,2.96)	0.19		
Vascular permeation (absent/present)	1.63(0.96,2.77)	0.07		
Postoperative Liver metastasis (negative/positive)	3.67(2.08,6.50)	<0.001	2.75(1.51,5.02)	<0.001
MSI2 expression (positive/negative)	2.64(1.46,4.78)	0.001	1.89(1.02,4.46)	0.04
NLK expression (positive/negative)	0.30(0.17,0.55)	<0.001	7.03(1.40,35.18)	0.02

### NLK promotes invasion and migration ability of PC cells through the activation of the EMT and PI3K-AKT-mTOR signaling pathway

**Fig. 3:**
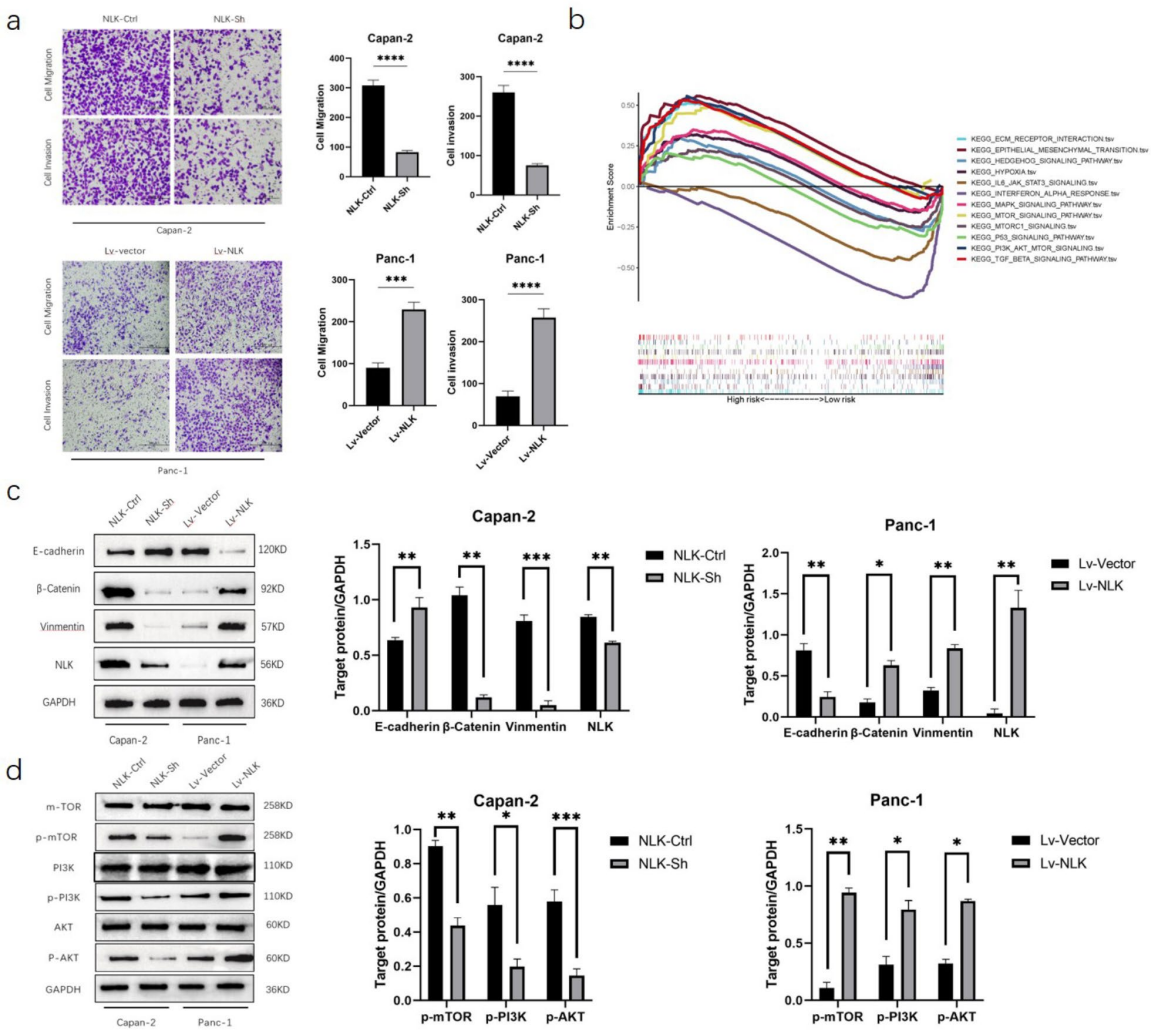
NLK promotes invasion and migration ability of PC cells through the activation of the EMT and PI3K-AKT-mTOR signaling pathway. (**a**) Transwell cell culture system showed the effect of NLK expression on tumor invasion and migration. (**b**) KEGG analyzed the functional enrichment of NLK expression between high and low groups. (**c**) Western Blot showed that the effects of NLK on EMT-related proteins. (**d**) Western Blot showed that the effects of NLK on PI3K/AKT/mTOR pathway proteins

To confirm the role of NLK in PC cell function, we transfected NLK-ShRNA (NLK-Sh) and negative control (NLK-Ctrl) in Capan-2 cells, and overexpressed NLK (Lv-NLK) and negative control (Lv-Vector) in Panc-1 cells. Transwell cell culture system showed that the invasive and migratory abilities of NLK-Sh transfected cells in Capan-2 cells were significantly reduced compared to the NLK-Ctrl group, while the invasive and migratory abilities of Lv-NLK overexpressing cells in Panc-1 cells were significantly increased compared to the Lv-Vector group (Fig. 3a). GSEA analysis of NLK in TCGA database showed significant enrichment in the EMT and PI3K-AKT-mTOR pathway (Fig. 3b). Western blot results showed that the expression of the EMT-related protein E-cadherin increased, while the expression of Vimentin and β-catenin decreased after knocking down NLK; the expression of E-cadherin decreased while the expression of Vimentin and β-catenin increased after overexpressing NLK (Fig. 3c). Similarly, compared to the control group, the expression of proteins in the PI3K-AKT-mTOR signaling pathway [p-PI3K (Tyr458), p-AKT (Ser473), and p-mTOR (Ser2448)] decreased after silencing NLK, while the expression of proteins in the PI3K-AKT-mTOR signaling pathway increased after overexpressing NLK (Fig. 3d).

**Fig. 4:**
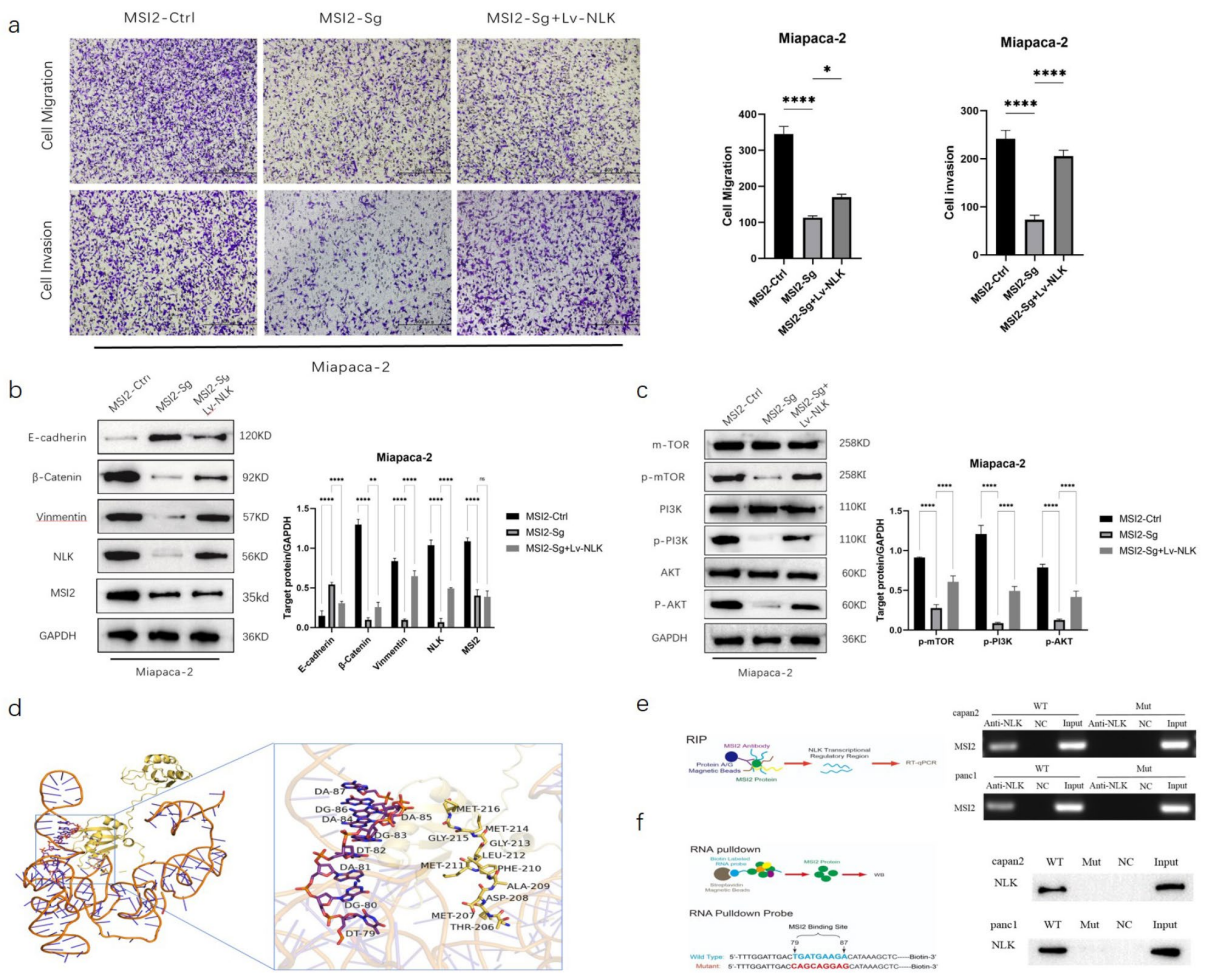
MSI2 directly binds to the translation regulatory region of NLK to activate the EMT and PI3K-AKT-mTOR signaling pathway in pancreatic cancer cells. (**a**) Transwell cell culture system showed the effect of MSI2 and NLK expression on tumor invasion and migration. (**b**) Western Blot showed that the effects of MSI2 and NLK on EMT-related proteins. (**c**) Western Blot showed that the effects of MSI2 and NLK on PI3K/AKT/mTOR pathway proteins. (**d**) Molecular docking predictions performed the interaction between MSI2 and NLK. (**e**) RIP indicated the specific binding of MSI2 to the translation regulatory region of the NLK gene. (**f**) RNA Pulldown demonstrated that sites 79-87nt of NLK could bind specifically to MSI2 protein

We further verified the role of MSI2 in the EMT and PI3K-AKT-mTOR signaling pathway of pancreatic cancer cells through rescue experiments. In Miapaca-2 cells, compared to MSI2-Ctrl, the invasive and migratory abilities of tumor cells were reduced in the MSI2-Sg group, but partially rescued after co-transfection with Lv-NLK (Fig. 4a). Western blot results showed that after interfering with MSI2 in Miapaca-2 cells, the expression of EMT-related protein E-cadherin increased, while the expression of Vimentin and β-catenin decreased, inhibiting the occurrence of EMT. After co-transfection with Lv-NLK, the expression of E-cadherin decreased, while the expression of Vimentin and β-catenin increased, the process of EMT was reversed (Fig. 4b); similarly, the protein expression levels of the PI3K-AKT-mTOR signaling pathway decreased after interfering with MSI2-Sg in Miapaca-2 cells, and the decreased protein expression levels of the PI3K-AKT-mTOR signaling pathway were reversed after co-transfection with Lv-NLK, while the total protein levels of PI3K, AKT, and m-TOR did not change (Fig. 4c). To further clarify the mechanism by which MSI2 regulates NLK, molecular docking predictions were performed. The result showed that the distance between the amino acid residues 201–250 of MSI2 and the nucleotides 79–87 of NLK are close, which meant that there is a large interaction trend and a high binding possibility between MSI2 and NLK (Fig. 4d). Further RIP experiments were down, followed by PCR amplification and agarose gel electrophoresis analysis, revealed that MSI2 specifically binds to the wild-type sequence of NLK mRNA at positions 79–87, but not to the mutant plasmid at positions 79–87, indicating the specific binding of MSI2 to the translation regulatory region of the NLK gene (Fig. 4e). RNA Pulldown experiments also confirmed that the wild-type probe of NLK mRNA at positions 79–87 specifically binds to MSI2, while the mutant probe of NLK mRNA at positions 79–87 does not, confirming that MSI2 can exert post-transcriptional regulation on NLK via the 79-87nt site of NLK mRNA (Fig. 4f).

### MSI2 regulates NLK to promote liver metastasis of pancreatic cancer in vivo

**Fig. 5:**
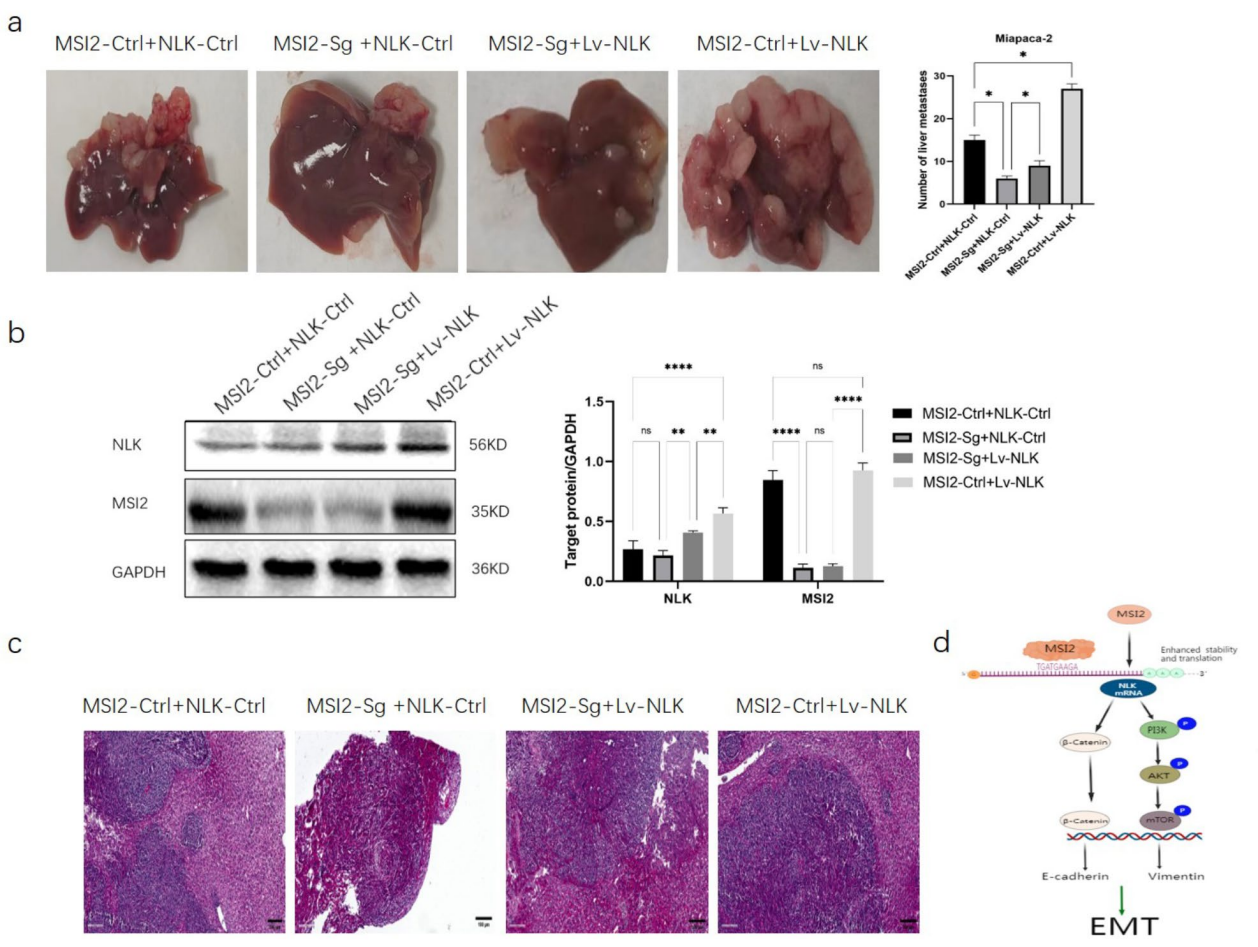
MSI2 regulates NLK to promote liver metastasis of pancreatic cancer in vivo. (**a**) Nude mouse transplantation tumor experiments constructed liver metastasis model of pancreatic cancer. (**b**) Western Blot showed that the protein level of NLK and MSI2 in pancreatic cancer tissues in nude mouse. (**c**) HE staining of liver metastatic tissues of pancreatic cancer. (**d**) Diagram of the study. MSI2 directly binds to the NLK mRNA to maintain its stability and promote the invasion and migration of pancreatic cancer cells, and NLK promotes pancreatic cancer progression through the EMT and PI3K/AKT/mTOR signaling pathway

To further verify the regulation of MSI2 and NLK on pancreatic cancer cells in vivo, we performed nude mouse transplantation tumor experiments. The results showed that compared to the MSI-Ctrl + NLK-Ctrl group, the number of liver metastases was significantly reduced in the MSI2-Sg + NLK-Ctrl group, while the restriction of liver metastasis was reversed in the MSI2-Sg + Lv-NLK group, and the highest number of liver metastases was observed in the MSI-Ctrl + Lv-NLK group (*p*=0.001) (Fig. 5a). The expression levels of NLK and MSI2 in pancreatic cancer tissues in nude mouse were verified by Western Blot (Fig. 5b). HE staining of liver metastatic tissues also showed that knocking down MSI2 effectively reduced the occurrence of liver metastasis, while upregulation of NLK promoted the occurrence of liver metastasis (Fig. 5c). Through in vivo and in vitro experiments, we revealed that MSI2 directly binds to the NLK mRNA to maintain its stability and promote the invasion and migration of pancreatic cancer cells, and NLK promotes pancreatic cancer progression through the EMT and PI3K/AKT/mTOR signaling pathway (Fig. 5d).

## Discussion

Pancreatic cancer is a leading cause of cancer death worldwide and its global burden has more than doubled over the past 25 years [26]. Further elucidating the mechanisms of pancreatic cancer occurrence and development is beneficial for improving the prognosis of pancreatic cancer patients. MSI2 is associated with the development of various solid tumors, but the mechanism of its binding with downstream genes as an RNA binding protein has not been fully studied. NLK, as an evolutionarily conserved serine/threonine protein kinase activated during mitosis, has been found to promote or inhibit different tumors, and its role in pancreatic cancer is still unclear. Study have shown that NLK overexpression in gallbladder cancer is significantly correlated with TNM stage and perineural invasion, leading to a worse prognosis [27]. In colorectal cancer, NLK levels are significantly elevated compared to adjacent tissues and are correlated with tumor size and depth of invasion [28]. In our study, the expression of MSI2 is positively correlated with NLK in pancreatic cancer. Multivariate analysis revealed that MSI2 and NLK were independent adverse indicators for the survival of pancreatic cancer patients, and their combined effect on the survival of pancreatic cancer patients has not been reported in previous studies.

EMT is considered to be the initiating factor for the transformation of benign to malignant tumors, and mediated malignant biological behavior involves multiple signaling pathways [29, 30]. EMT plays an important role in various malignant biological behavior of pancreatic cancer. Numb-PRRL amplifies EMT-activating factors in pancreatic cancer [31]. GINS can downregulate E-cadherin through specific activation of the ERK/MAPK signal, promoting EMT in pancreatic cancer [32]. Our results demonstrate that NLK can promote the occurrence and development of EMT in pancreatic cancer cells. However, due to different types of cancer and their associated cellular environments, the role of NLK may vary or even be opposite in different types of cancer [33]. Reports suggested that NLK overexpression can inhibit the occurrence of EMT and subsequently inhibit the proliferation and migration of non-small cell lung cancer (NSCLC) by affecting E-cadherin protein expression [34, 35]. The silence of MSI2 repressed extrahepatic cholangiocarcinoma cell migration and invasion by inhibiting epithelial-mesenchymal transition [36]. Besides, MSI2 might enhance invasion of hepatocellular carcinoma by inducing epithelial-mesenchymal transition [37]. Building on our previous research, this study further confirms that MSI2 can affect EMT by regulating NLK, and both of them together promote the deterioration of the prognosis of pancreatic cancer patients, which also provides a potential target for EMT-targeted therapy in pancreatic cancer.

The PI3K/AKT signaling pathway is a key signaling pathway that induces tumor cell growth and invasion, and plays a crucial role in EMT initiation [33, 38, 39]. In prostate cancer, MSI2 can bind to the 3’-UTR region of androgen receptor (AR) mRNA, enhancing its mRNA stability and translation activity, and regulating the PI3K/AKT/mTOR pathway [40]. In addition, microRNA-149 could suppress the malignant phenotypes of ovarian cancer via downregulation of MSI2 and inhibition of PI3K/AKT pathway [41]. The regulation of PI3K/AKT/mTOR signaling by MSI2 and NLK in pancreatic cancer is still unclear. Our study confirmed that overexpression of NLK increased the expression of p-PI3K (Tyr458), p-AKT (Ser473), and p-mTOR (Ser2448) in vitro, while knockout of NLK suppressed the PI3K/AKT/mTOR signaling pathway and inhibited EMT in pancreatic cancer cell lines.

This study has some limitations. The study found that different subtypes of MSI2 can affect the prognosis of breast cancer [9], but we did not distinguish the effect of the MSI2 subtype on pancreatic cancer progression. In animal experiments, the mortality rate of mice in MSI2-Ctrl + Lv-NLK group was too high, although the number of animals in other groups was normal, but the reliability of animal experimental data was affected. mTOR can catalyze two multiprotein complexes, mTORC1 and mTORC2 [20]. Whether NLK promotes the expression of mTORC2 through p-AKT (Ser473) will be an important point to explore in the future.

In summary, our study revealed that MSI2 can specifically bind to the translational regulatory region of the 79-87nt of NLK mRNA, exerting post-transcriptional regulatory effects. The high expression of NLK activates the PI3K/AKT/mTOR and EMT signaling pathways, promoting the invasion and migration of pancreatic cancer cells. We have identified a novel therapeutic target for pancreatic cancer and provided help for the clinical treatment of pancreatic cancer.

### Electronic supplementary material

Below is the link to the electronic supplementary material.

Supplementary Material 1

Supplementary Material 2

## Data Availability

No datasets were generated or analysed during the current study.
