# Supplementary material for: MSI2 regulates NLK-mediated EMT and PI3K/AKT/mTOR pathway to promote pancreatic cancer progression
Source: Cancer Cell Int. 2024 Aug 3;24:273. doi: 10.1186/s12935-024-03444-9 (PMC11297748; doi:10.1186/s12935-024-03444-9)
Supplement: Supplementary file 1 — Supplementary Material 1 [file 12935_2024_3444_MOESM1_ESM.docx]

**Supplementary information**

**Table S1. Primer sequence of PCR**

| Gene |  | Sequence |
| --- | --- | --- |
| MSI2 | Forward | 5’-AGACCTCACCAGATAGCCTTAG-3’ |
|  | Reverse | 5’-CCACTACTGTGTTCGCAGATAA-3’ |
| PREPL | Forward | 5’-AGAAGGTTGTTGCTTGGTTCG-3’ |
|  | Reverse | 5’-TCAATGAAGGGCTGGTCTAACT-3’ |
| COIL | Forward | 5’-GAGACGGTTAGGCTACGGC-3’ |
|  | Reverse | 5’-GACGACTCGGCATCTGTTCAA-3’ |
| NSF | Forward | 5’-AAGCGGCAAGATGTCCTACAG-3’ |
|  | Reverse | 5’-CCACCGATGGATGTGTCTTCA-3’ |
| TRIM37 | Forward | 5’-TATGGAGAAATTGCGGGATGC-3’ |
|  | Reverse | 5’-GTCAGCCAGCGCCTAATACAG-3’ |
| SLC36A4 | Forward | 5’-CGCGAGGAGCTAGATATGGAT-3’ |
|  | Reverse | 5’-TGGAAGTCCTAAAAGGCCAGT-3’ |
| ZNF24 | Forward | 5’-CTGATGGCGAAGAGGGATCAA-3’ |
|  | Reverse | 5’-CCAGCACTACCAGCTCCAAG-3’ |
| KIF3B | Forward | 5’-TGGATGTGGATGTTAAGCTGGG-3’ |
|  | Reverse | 5’-TCGGAACGTCTCATCGTACAG-3’ |
| C17orf80 | Forward | 5’-AATCCCACTTGCCATACTGTAAG-3’ |
|  | Reverse | 5’-ACTGTCTGTTCTGGTTTGTCCA-3’ |
| DYNLL2 | Forward | 5’-ACCCTACCTGGCATTGTATCG-3’ |
|  | Reverse | 5’-AGCCTGACTTGAAGAGGAGGA-3’ |
| GSTCD | Forward | 5’-ACCATCCCTTTGGCTATTGAGA-3’ |
|  | Reverse | 5’-TTCTGCCTGCGGAGTTTATCA-3’ |
| NLK | Forward | 5’-CGCAAAAATGATGGCGGCTTA-3’ |
|  | Reverse | 5’-CCCAGGGTTTAACATGGCTG-3’ |
| VEZF1 | Forward | 5’-GGACAGCTATCACCTGAGGC-3’ |
|  | Reverse | 5’-GCGATGGTAGAGATAAGGGGAA-3’ |
| CALCOCO2 | Forward | 5’-ATTTCATCCCTCGTCGAAAGGA-3’ |
|  | Reverse | 5’-TGAAGGTGTAATACTCACGGGTT-3’ |
| COX11 | Forward | 5’-TGGAGGTGCGTTCCTTTCTG-3’ |
|  | Reverse | 5’-GAAACGGCTCTACCCTCTCTG-3’ |
| PPM1D | Forward | 5’-CTGTACTCGCTGGGAGTGAG-3’ |
|  | Reverse | 5’-GTTCGGGCTCCACAACGATT-3’ |
| GAPDH | Forward | 5’-CATGAGAAGTATGACAACAGCCT-3’ |
|  | Reverse | 5’-AGTCCTTCCACGATACCAAAGT-3’ |

**Table S2 The corporation and scale of primary antibodies**

| Gene | Corporation | Scale |
| --- | --- | --- |
| MSI2 | Abcam | IHC:1:200 WB:1:2000 |
| NLK | Cell Signaling Technology | IHC:1:200 WB:1:2000 |
| E-cadherin | Abcam | IHC:1:200 WB:1:2000 |
| β-catenin | Proteintech | IHC:1:100 WB:1:2000 |
| Vimentin | Proteintech | IHC:1:100 WB:1:2000 |
| PI3K | Bimake | IHC:1:200 WB:1:1000 |
| AKT | Bimake | IHC:1:200 WB:1:1000 |
| m-TOR | Bimake | IHC:1:200 WB:1:1000 |
| p-PI3K | Cell Signaling Technology | IHC:1:100 WB:1:2000 |
| p-AKT | Cell Signaling Technology | IHC:1:100 WB:1:2000 |
| p-mTOR | Cell Signaling Technology | IHC:1:100 WB:1:2000 |
| GAPDH | Proteintech | WB:1:2000 |

**Table S3 The sequence of Si-RNA**

| Gene | Si-RNA sequence 5'- 3' | The siRNA- Ctrl sequence 5'-3' |
| --- | --- | --- |
| MSI2 | GACCCAGCAAGUGUAGAUATT | UUCUCCGAACGUGUCACGUTT |
| NLK | CGCGTCCCCGCAAGAGGCTAGGCT | CAAAGCAAGATTAGTACCTGGACC |
