# Supplementary material for: MSI2 regulates NLK-mediated EMT and PI3K/AKT/mTOR pathway to promote pancreatic cancer progression
Source: Cancer Cell Int. 2024 Aug 3;24:273. doi: 10.1186/s12935-024-03444-9 (PMC11297748; doi:10.1186/s12935-024-03444-9)
Supplement: Supplementary file 2 — Supplementary Material 2 [file 12935_2024_3444_MOESM2_ESM.docx]

**Figure S1 RNA expression levels of 15 MSI-related genes**


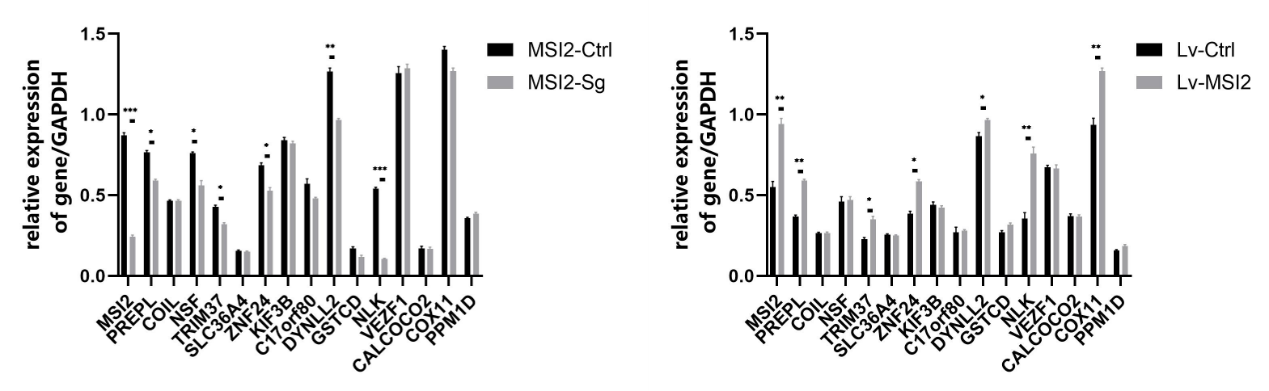


Figure S1: qRT-PCR showed that the RNA level changes of 15 MSI-related genes in MSI2 knockdown stable cell lines established in Capan-2 cells and MSI2 overexpression stable cell lines constructed in Panc-1 cells.
